# Optimized transesterification of unrefined palm and waste cooking oil blend to biodiesel using cement kiln dust catalyst

**DOI:** 10.1038/s41598-025-11752-x

**Published:** 2025-07-28

**Authors:** Mahmoud S. Hefney, Mai O. Abdelmigeed, Tamer S. Ahmed, Ibrahim M. Ismail

**Affiliations:** 1 Zewail City of Science and Technology, 6Th of October City, Giza, 12578 Egypt; 2 Chemical Engineering Department, Faculty of Engineering, Cairo University, Giza, 12613 Egypt

**Keywords:** Transesterification, Biodiesel, Cement kiln dust (CKD), Factorial design, Optimum conditions, Engineering, Chemical engineering

## Abstract

Biodiesel is a sustainable alternative to fossil fuels. However, biodiesel’s economic feasibility for mass-production remains a challenge. This study investigates the use of cement kiln dust (CKD) waste as a heterogeneous catalyst for the transesterification of a mixed oil made from unrefined palm oil and waste cooking oil (WCO) to increase the cost-effectiveness. To enhance the activity of the CKD, it was treated at optimized conditions of 850 °C for 2 h. The optimal blend (60 wt% WCO) for transesterification using CKD was determined through a comparative analysis of different ratios. The WCO was first upgraded through acid-catalyzed esterification to reduce free fatty acids < 1%. The effects of reaction time, temperature, catalyst loading, and methanol-to-oil ratio on biodiesel using CKD were studied using a full factorial 2^n^ design and response surface methodology. Optimized combinations of reaction parameters were presented that give near complete conversion, which is much higher compared to the literature. These combinations can work under different manufacturing or market challenges. The produced biodiesel meets ASTM standards. This work demonstrates the potential for using low-value waste as a catalyst and low-value raw materials for biodiesel production and highlights the importance of optimizing process parameters for economic viability.

## Introduction

In recent years, there has been a heightened focus on research pertaining to renewable energy and the exploration of novel energy sources. This increased attention can be attributed primarily to concerns regarding economic stability, energy security, and environmental sustainability. In an effort to diminish reliance on fossil fuel sources and promote ecological sustainability, numerous nations have pledged to adopt renewable energy solutions and/or to reduce greenhouse gas emissions at both national and international levels^1,2^. Biodiesel is regarded as one of the most viable renewable energy sources for transportation, as it can seamlessly substitute mineral diesel within the current technological frameworks without necessitating significant modifications. This compatibility has prompted numerous countries to implement legislation aimed at promoting the utilization and blending of biodiesel with mineral diesel^3^.

Biodiesel is composed of alkyl esters produced mainly from plant-based oils (edible and non-edible) or animal fats^4^ and has fuel properties similar to diesel fuel, which makes it usable as an alternative fuel^5^. The primary obstacles hindering the advancement of biodiesel production are twofold: first, the cost and accessibility of feedstock; and second, the technical and economic considerations associated with the conversion process of oils into biodiesel^6^. Utilizing inexpensive feedstock and repurposing waste cooking oils and animal fats presents a viable strategy for mitigating feedstock expenses^7^. Also, Enhancements in processes and optimization strategies contribute to the reduction of costs associated with the biodiesel conversion process^8^. The synthesis of biodiesel primarily occurs through the transesterification of oils, which represents the predominant and most widely utilized method in the production process of biodiesel^9^.

In the context of feedstock materials for biodiesel production, palm oil presents a viable alternative owing to its accessibility and affordability, particularly in the countries where it is cultivated. It can be utilized in both refined and unrefined forms. Despite the advantages of availability and cost-effectiveness associated with palm oil, utilizing unrefined palm oil or by-products from the palm oil industry, such as palm oil sludge, is significantly more advantageous for food security and waste management considerations^10^. Following the extraction of palm oil and palm kernel oil, the residual biomass can be utilized for the production of alternative biofuels, including biogas, bio-hydrogen, and bioethanol. The remaining solid materials may subsequently be repurposed as fertilizers or as feed for livestock^11^. From the life cycle scheme for biodiesel production from palm by Pleanjai and Gheewala^12^, It can be noted that various extraction and production processes require multiple energy inputs and yield several valuable by-products. Consequently, unrefined palm oil, along with any waste materials generated from this industry, presents a viable option for biodiesel production. Another commonly utilized raw material in the biodiesel sector is waste cooking oil (WCO), which refers to any vegetable oil that has been collected after its use in cooking and/or frying. Although WCO possesses less desirable characteristics compared to its original virgin oil for biodiesel production, it is regarded as a highly cost-effective raw material, as it can often be acquired at little to no cost. The improper disposal of WCO, whether into drainage systems or the environment, poses significant environmental challenges, including water and soil contamination, as well as complications in wastewater treatment. The substantial growth of the human population is likely to exacerbate the generation of WCO, as its production is closely linked to the increasing demand for food. Overall, the utilization of WCO for biodiesel production can yield positive contributions to waste management, food security, environmental sustainability, and energy security.

Although homogeneous catalysts possess certain advantages, there is presently significant attention directed towards the chemical synthesis of heterogeneous catalysts for the production of biodiesel through transesterification processes^13^. This interest gets bigger and bigger to reach a way of commercializing them in the biodiesel industry on a large scale^14^. This transesterification method presents several advantages, including the reusability of catalysts, reduced purification needs, ease of separation, capability to process oils with elevated free fatty acid (FFA) and water content, as well as the generation of products that are nearly pure^15^. Heterogeneous catalysts contain active components capable of facilitating both esterification and transesterification reactions. An example of such a catalyst is calcium oxide (CaO), which functions as a metal oxide catalyst^16^. They can also feature high surface area carrier structures like metal–organic frameworks (MOFs) impregnated with NaOH^17,18^. Additionally, catalysts with 3D hierarchical porous composites structures that can also combine mesoporous and microporous features are known for their enhanced mass transfer capabilities and increased exposure of active sites, contributing to their high catalytic efficiency^19–22^. Notwithstanding the numerous advantages associated with the utilization of these catalysts, certain limitations persist, including the requirement for elevated operating conditions (e.g., agitation speed, temperature, etc.) and the comparatively reduced catalytic activity of certain heterogeneous catalysts in relation to their homogeneous counterparts. These limitations can be effectively addressed or alleviated through the optimization of reaction conditions and the parameters that influence the transesterification process. Furthermore, the economic viability of the process has garnered significant interest among researchers, resulting in the development of new generations of more cost-effective heterogeneous catalysts derived from waste materials, which may offer both environmental and economic advantages^23^.

**Fig. 1:**
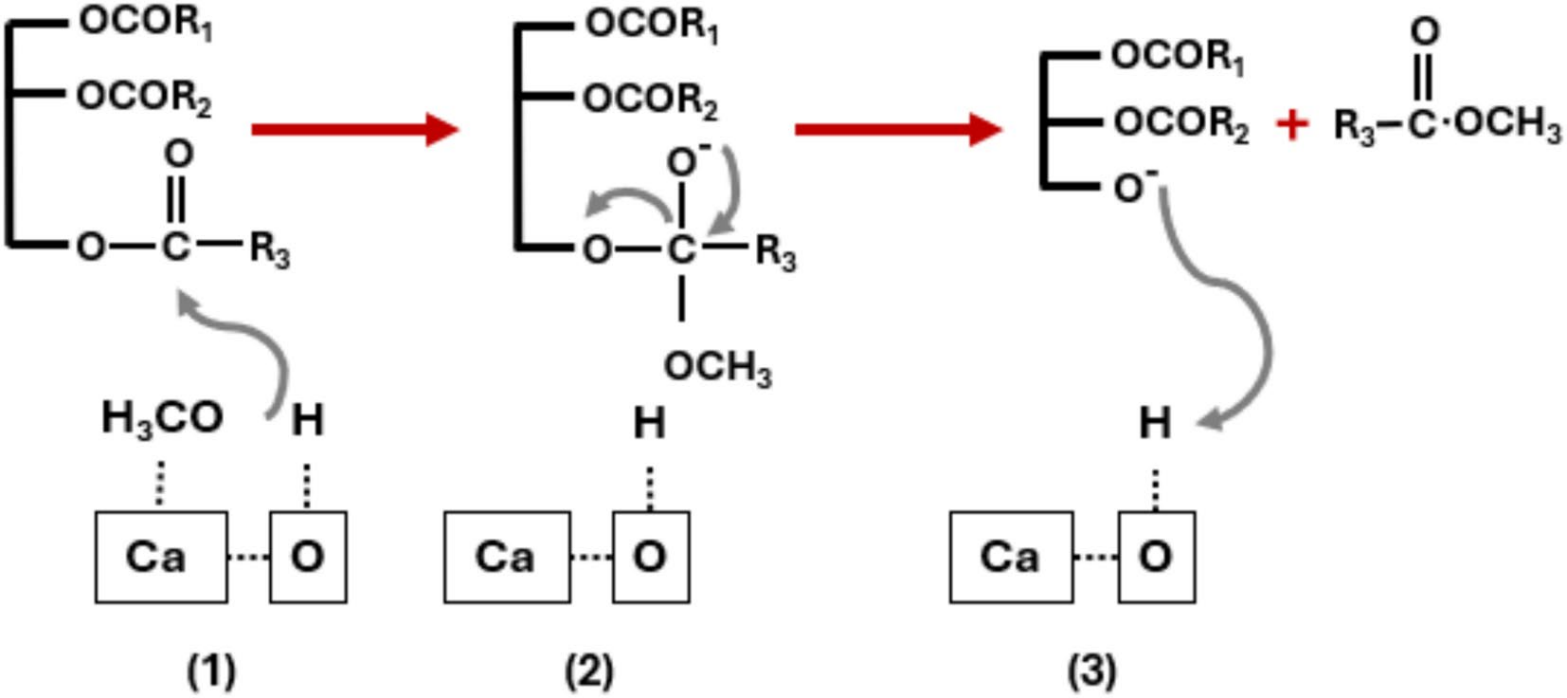
Transesterification reaction mechanism using CaO as an active component of catalyst^35^.

Cement kiln dust (CKD), marble dust, fish bones, and eggshells are representative examples of waste materials that contain significant amounts of active constituents, particularly calcium oxide (CaO)^24,25^. Consequently, harnessing the potential of waste materials may yield significant advantages, including the improvement of the economic viability of large-scale biodiesel production and the effective management of these waste products within industrial applications. It is essential to implement treatment methods to convert and activate waste materials into functional components, such as calcium oxide (CaO). Figure 1 illustrates the mechanism by which the transesterification reaction transpires, utilizing either pure CaO or waste-derived CaO as a catalyst for the synthesis of methyl esters (biodiesel). In their research, Buasri et al. investigated the transesterification process of palm oil employing chicken and duck shells as catalytic agents^26^. The waste materials underwent a treatment process involving calcination for a duration of four hours at a temperature of 900 °C, followed by the optimization of transesterification conditions to achieve maximum conversion rates. The optimal parameters identified included a methanol-to-oil molar ratio of 9:1, a reaction temperature of 65 °C, a catalyst loading of 20%, and a reaction duration of four hours. Under these specified conditions, the conversion rates attained were 92.92% for duck shells and 94.49% for chicken shells. Furthermore, the catalysts demonstrated the potential for reuse; however, a decline in conversion efficiency was observed after four cycles. Additionally, other researchers have suggested the utilization of fish scales as a catalyst source (specifically for CaO) in the synthesis of biodiesel^27^. In order to enhance the reaction conditions and evaluate the catalytic activity, a catalyst derived from calcined fish bones, prepared at a temperature of 900 °C, was employed with a consistent loading of 10% relative to the mass of palm oil during transesterification experiments. The optimal process conditions achieved a conversion rate of 77.2% at a reaction temperature of 65 °C, a reaction duration of 4 h, a calcination period of 3 h, and a methanol-to-oil molar ratio of 12:1^28^.

Countries engaged in cement production generate substantial quantities of cement kiln dust (CKD). For instance, Egypt produces approximately 3 million tons annually, which is typically regarded as a byproduct or waste material^29^. Unlocking the potential of using CKD is essential from both environmental and economic aspects^30^. The industrial repurposing of cement kiln dust (CKD) has been the subject of extensive research, which indicates its potential applications in improving the properties of cement, activating slag, manufacturing glass, and producing environmentally sustainable products, including biodiesel and wollastonite^28^.

Samidi^31^ developed two CKD-based catalysts for the transesterification of WCO. The first catalyst was synthesized by impregnating CKD with potassium hydroxide (KOH), while the second was prepared using a mixture of water and methanol. Prior to the transesterification process, both catalysts underwent calcination at a temperature of 650 °C for a duration of three hours. The findings of the study indicated that the CKD-KOH catalyst exhibited superior catalytic activity compared to the CKD catalyst prepared with methanol and water. Al-Sakkari and coworkers^23,28^ conducted a study on the synthesis of biodiesel from virgin soybean oil utilizing cement kiln dust (CKD) as a heterogeneous catalyst. Their research focused on various factors influencing biodiesel production, including reaction time, the molar ratio of methanol to oil, and catalyst concentration, while also identifying the optimal reaction conditions. Under fixed parameters of a mixing speed of 800 rpm and a temperature of 65 °C, the optimal conditions were determined to be approximately 6 h of reaction time, a catalyst loading of 2% relative to the mass of oil, and a methanol-to-oil molar ratio of 15:1. Stability assessments of the CKD catalyst indicated that it is not reusable due to issues of leaching and deactivation. The reusability of CKD catalysts is typically influenced by several factors, including their stability, leachability, the specific reaction conditions employed, and the degree of catalytic activity loss during subsequent uses^32^. Typically, prior efforts to utilize cement kiln dust (CKD) in biodiesel production have resulted in conversions that fall short of complete conversion under standard reaction conditions^23,28,33,34^. This phenomenon may be linked to the specific treatment conditions applied to the CKD. Consequently, this presents an opportunity for enhancement and the exploration of the material’s potential.

The economic viability of utilizing waste cooking oil (WCO) as a feedstock for biodiesel production is noteworthy. Nonetheless, the quality of the biodiesel produced is significantly influenced by the quality of the WCO utilized^36^. To address this concern, one potential strategy involves blending WCO with a cost-effective and readily accessible oil, such as unrefined palm oil. This study examines the efficacy of a CKD heterogeneous catalyst in achieving near-complete conversion of a blend of WCO and unrefined palm oil into biodiesel. The CKD catalyst underwent treatment under optimized conditions to enhance its catalytic activity, while various blending ratios of the two oils were systematically evaluated to determine the optimal composition for the transesterification process. Factors influencing biodiesel production, including reaction time, temperature, catalyst loading, and the methanol-to-oil molar ratio, were analyzed using a factorial design methodology and subsequently optimized through response surface methodology. Following the optimization process, the principal properties of the produced biodiesel were assessed and compared against ASTM standards.

The present study investigates the feasibility of employing low-cost waste materials, specifically cement kiln dust (CKD), as a catalyst in conjunction with waste cooking oil (WCO) and unrefined palm oil as feedstocks, to improve the economic viability of large-scale biodiesel production. This research underscores the necessity of optimizing process parameters to achieve cost-effective biodiesel synthesis. A notable contribution of this study is the markedly enhanced catalytic activity of the CKD catalyst, which surpasses findings reported in existing literature. This improvement is ascribed to a specialized pre-treatment of the CKD, aimed at augmenting its efficacy in the transesterification process.

## Materials and methodology

### Materials

#### Feedstock (mixed oils)

The feedstock utilized in the current investigation comprises a blend of waste cooking oil (WCO) and unrefined palm oil, formulated at an optimized ratio as delineated in the Methodology section, to achieve enhanced product characteristics. The unrefined palm oil employed in this study exhibits inferior cold flow properties and a higher acid value in comparison to virgin refined palm oil, which is predominantly utilized for culinary applications. The WCO component of the mixture, as indicated on the product label, consists of an equal proportion of 50% soybean oil and 50% sunflower oil, both of which are commonly used for frying and cooking. The selection of unrefined palm oil for biodiesel production was primarily motivated by concerns related to food security. Additionally, the incorporation of WCO as a feedstock addresses environmental challenges associated with waste disposal and underscores the issues arising from the excessive use of cooking oils. The physical properties of the palm oil analyzed in this study are presented in Table 1, while its compositional analysis is detailed in Table 2. The fatty acid methyl ester (FAME) composition was determined using gas chromatography-mass spectrometry. Conversely, the properties of the WCO are outlined in Table 3.

**Table 1:** Physical and chemical properties determined for palm oil used.

Property (unit)	Value
Density at 20 °C (g/mL)	0.89
Dynamic viscosity at 30 °C (cP)	57.8
Acid value (mg KOH/g Oil)	2.8
Water content wt %	< 0.01%
Color	Pale yellow
Odor	Odorless
Slip melting point (°C)	34

**Table 2:** Fatty acid composition of palm oil used.

Fatty acid (carbon number)	Percentage %
Myristic acid (14:0)	1
Palmitic acid (16:0)	44
Stearic acid (18:0)	4.5
Oleic acid (18:1)	39
Linoleic acid (18:2)	9.5
Linolenic acid (18:3)	0.4
Free fatty acids	1.4

**Table 3:** Properties of WCO used.

Property	Value
Density at 20 °C (g/mL)	0.902
Dynamic viscosity at 20 °C (cP)	45.1
Acid value mg KOH/g oil	18.35
Water content wt %	< 0.01%
Color	Light brown
Odor	Unpleasant
Slip melting point (°C)	36

#### Cement kiln dust (CKD)

The catalyst employed in the present investigation was derived from waste CKD sourced from the Tura Cement Factory, situated in Helwan, south of Cairo, Egypt. This facility is recognized as one of the largest cement production plants in the country. To eliminate moisture from the catalyst and facilitate the conversion of calcium carbonate into calcium oxide—an effective agent for catalyzing the transesterification reaction—the CKD underwent calcination under optimized conditions at 850 °C for a duration of two hours. The calcination process at this temperature induces significant transformations; specifically, the calcium carbonate present in CKD decomposes into calcium oxide (CaO), which serves as a highly active catalytic site due to its pronounced basicity, thereby promoting reactions such as transesterification. Furthermore, the calcination alters the structural characteristics of the material, potentially enhancing the surface area and pore volume, which in turn increases the availability of active sites for interaction with reactants. This process also results in the formation of mixed oxides, including calcium silicates, which introduce additional active sites, albeit with a lower basicity compared to pure CaO. The selected temperature was intended to augment the catalyst’s activity and was higher than that utilized by Al-Sakkari et al.^23^ (740 °C).

The CKD samples used in this study are identical to those previously characterized and utilized by Al-Sakkari et al.^23^ Building upon their work, our research aimed to enhance the catalytic performance, achieve complete conversion, and introduce the use of waste cooking oil (WCO) as a feedstock in place of virgin oil. This approach supports greater sustainability in biodiesel production.

Approximately 56% of the CKD particles are classified as being larger than 75 microns and smaller than or equal to 90 microns, while the remaining particles fall within the range of 63 to 75 microns. The specific surface area of the CKD utilized in this study is measured at 17.8 m^2^/g. Furthermore, the CKD analyzed contains active components constituting approximately 54% of its total weight, with calcium oxide accounting for 45.89% and other alkalis comprising around 8% of the overall weight.

#### Other chemicals

The chemicals utilized in various phases of the biodiesel production process were procured from Sigma-Aldrich and employed without additional purification. These included potassium hydroxide (KOH, 99% purity), sulfuric acid (H₂SO₄, 99% purity), and calcium chloride (CaCl₂, 99% purity).

### Methodology

#### Determination of the optimum palm oil to WCO ratio to be used with the CKD catalyst

Seven distinct palm oil to waste cooking oil (WCO) weight percentage ratios (100:0, 80:20, 60:40, 50:50, 40:60, 20:80, and 0:100) were prepared through the blending of the two oils, as illustrated in Figure S-1 of the supplementary document. These seven blends underwent transesterification to produce biodiesel via a two-step process. Initially, an acid-catalyzed esterification was performed on the high free fatty acid (FFA) content of the collected WCO to reduce its acidity prior to blending. Subsequently, a homogeneous base-catalyzed transesterification was conducted on the blended samples to facilitate their conversion into biodiesel. The utilization of homogeneous acid and base catalysts was intended to ensure that all seven blends achieved nearly complete conversion. The cold flow properties, specifically the cloud and pour points, as well as the acid values of the biodiesel produced from each blend, were assessed and compared. Ultimately, a comparative analysis was conducted to identify the optimal blend for further transesterification process optimization utilizing the CKD catalyst.

In the initial phase of the process, acid-catalyzed esterification was employed to reduce the free fatty acid (FFA) content of the collected waste cooking oil (WCO) to below 1%. A 250 ml glass spherical bottom flask was utilized, into which 100 g of the collected WCO was introduced and heated to 65°C on a hot plate. Subsequently, a solution comprising a mixture of methanol and sulfuric acid was added to the flask, with sulfuric acid serving as the catalyst at a loading of 3.5% relative to the WCO. Methanol was used at a ratio of 52:1 to the oil, equating to approximately 185 ml of methanol. The mixture was subjected to moderate stirring via a magnetic stirrer for a duration of one hour, maintaining the temperature at 65°C. A water condenser was employed to recirculate the condensed vapors, primarily methanol, back into the flask, thereby ensuring a closed system. Following the reaction, unreacted methanol was separated from the esterified WCO, and any excess methanol remaining in the oil was eliminated by heating the oil to 100°C for one hour. The acid value was subsequently measured to confirm the reduction in FFA content.

In the second phase of the experiment, which involved base-catalyzed transesterification, 20 g of each of the seven blends of palm oil and esterified waste cooking oil (WCO) were subjected to a reaction at a temperature of 65 °C, with moderate stirring employed to mitigate the risk of soap formation. Potassium hydroxide (KOH) served as the homogeneous base catalyst, and methanol was incorporated into all seven experimental setups. The KOH was utilized at a concentration of 1% relative to the 20 g of feedstock, while the methanol-to-oil ratio was maintained at 6:1, corresponding to 4.5 g of KOH. Following a one-hour reaction period, the resulting biodiesel and glycerol were subjected to heating to eliminate excess methanol, after which they were separated using a separating funnel. Subsequent to the separation of biodiesel, a water washing procedure was conducted to eliminate any residual catalyst, methanol, and other impurities. The biodiesel samples were then dried with anhydrous calcium chloride (CaCl2). These biodiesel samples were subsequently utilized in a comparative analysis aimed at identifying the optimal weight percentage ratio of palm oil to WCO for the purpose of optimizing the transesterification process using CKD catalyst.

#### Factorial design of experiments

A full factorial 2^n^ design was employed to optimize the n factors, or independent variables, that influence the synthesis of biodiesel. This design facilitated the execution of all transesterification experiments systematically, thereby yielding results that contribute to the optimization of reaction conditions. The four independent factors selected for this study include: × 1: time (ranging from 1 to 6 h), × 2: methanol to oil molar ratio (ranging from 12 to 18), × 3: catalyst loading (ranging from 1 to 3% of the total oil weight), and × 4: temperature (ranging from 45 to 65 degrees Celsius). The objective of the current study is to enhance the parameters established by Al-Sakkari.^23^, whose previous research focused on three parameters: reaction time (2–6 h), methanol-to-oil molar ratio (9–15), and catalyst loading (2–5 wt.%). In this study, we expanded the investigation to include a fourth parameter, temperature, in order to achieve a more comprehensive understanding of the reaction dynamics. The methanol-to-oil molar ratio was increased to a maximum of 18 to improve conversion efficiency, which was found to facilitate the transesterification reaction more effectively. This enhancement subsequently reduced the required catalyst loading, thereby improving cost efficiency in both the reaction process and the subsequent separation stages. The chosen reaction time range of 1 to 6 h enables the capture of the entire kinetic profile, from the initial reaction phase (1 h) to near-complete conversion (6 h), which is essential for process optimization. Catalyst loading was optimized to a range of 1–3%, demonstrating efficiency by balancing reaction effectiveness with the minimization of catalyst waste. In terms of temperature, a maximum of 65°C was maintained to prevent excessive methanol evaporation, ensuring an efficient reaction, while a minimum temperature of 45°C was established to effectively initiate the transesterification process. Collectively, these modifications enhance conversion efficiency, reduce costs, and align with optimal ranges reported in prior studies^23,26–28,32,34^.

The response value is the conversion percentage of the used mixed oils to methyl esters (biodiesel). There is a coding for each factor based on the chosen values for each one. These codes are two main levels: low (− 1) and high (+ 1), while center points took the code of (0). 16 factorial points, 8-star points, and 6-center points were done for the current study in the form of transesterification experiments. Center points are repeated six times to calculate the standard deviation of experiments.

#### Biodiesel synthesis (CKD-catalyzed transesterification)

Spherical bottom flasks with capacities of 250 ml and 500 ml were employed for the reactions. The majority of the apparatus utilized in the two-step transesterification process was also applicable to the CKD-catalyzed reaction. For the reaction, thirty grams of a blend of unrefined palm oil and waste cooking oil, at the optimal ratio determined from the comparative study of the two-step transesterification (ratio of 2:3), were heated in a round bottom flask to the designated temperatures of 45, 55, and 65 °C, as dictated by the factorial design. Subsequently, CKD was introduced into the flask at varying catalyst loadings of 1%, 2%, and 3% relative to the weight of the feedstock oil, and the mixture was agitated using a magnetic stirrer. Methanol was incorporated at different methanol-to-oil ratios of 12:1, 15:1, and 18:1, and thoroughly mixed with the other components of the reaction. The stirring speed was calibrated to 850 rpm to induce turbulence within the flasks and mitigate mass transfer limitations. The reactions were conducted for durations of 1, 3.5, and 6 h. Following each experimental run, CKD and all suspended solid impurities were removed from the mixture through filtration. The resulting filtrate, which contained the biodiesel phase, glycerol phase, and unreacted methanol in both phases, was subsequently heated at 100 °C for one hour in an oven to eliminate methanol from the mixture and facilitate the separation of the two phases. The mixture was then transferred into a separating funnel to allow glycerol to settle at the bottom, with biodiesel (methyl esters) remaining on the surface. Glycerol was extracted from the biodiesel and weighed to determine the reaction conversion percentage. In the transesterification process, the reaction of one mole of oil with three moles of methanol yields one mole of glycerol and three moles of methyl esters. Consequently, Eq. (1) delineates the methodology employed to estimate the yield of each conducted reaction.

1 $${\text{Yield}}\;\left( {{\text{conversion}}\;\% } \right) = \frac{{{\text{wt}}\left( {\text{p}} \right)}}{{{\text{wt}}\left( {\text{c}} \right)}}*100$$

where wt(p) is the weight of produced glycerol and wt(c) is the weight of glycerol at 100% conversion.

#### Statistical analysis and RSM

After conducting a factorial design and calculating the conversion percentages (responses) of the transesterification reactions, a regression equation was formulated to establish a relationship between the responses and the selected input variables. The significance of the coefficients of this regression equation was assessed using the t-test. The validity of the regression equation was evaluated through the determination coefficient (R^2^). Excel software facilitated the development of a linear model based on the factorial design data, while Design-Expert software (version 13.0.5.0) was employed to create a quadratic model from the same dataset. A comparative analysis of the determination coefficients of both models was performed to ascertain the more effective model for optimizing the reaction conditions. Response surface methodology (RSM) served as the primary optimization tool, enabling the establishment of a functional relationship and the generation of contour plots and response surfaces that depict the influence of the input variables on the conversion percentage.

#### Product characterization

##### Gas chromatography analysis for purity determination of FAMEs

Gas chromatography analysis was employed to ascertain the fatty acid profiles of palm oil and two distinct biodiesel samples, both of which exhibited nearly complete conversion. Sample (1) was synthesized under conditions of 65 ºC for 3.5 h, utilizing a methanol-to-oil ratio of 15:1 and a CKD loading of 3%. In contrast, sample (2) was produced at 60 ºC for 4.5 h, with a methanol-to-oil ratio of 17:1 and a CKD loading of 2.7%. The reaction parameters for sample (2) were derived from the response surface methodology and regression analysis, which identified them as the optimal conditions for the reaction. Conversely, the conditions for sample (1) were based on a previously established experimental design that ensured complete conversion. The identification of fatty acid methyl ester (FAME) composition was conducted using Gas Chromatography-Mass Spectrometry (GC–MS) with a silica capillary column (DB-5) characterized by an internal diameter of 0.32 mm and a length of 60 m. The column temperature was initially set at 150 ºC for one minute, followed by a gradual increase to 240 ºC over a duration of thirty minutes. Helium served as the carrier gas, maintained at a flow rate of 1 ml per minute. Internal standards were utilized to convert the generated peaks into quantitative fractions of the fatty acid composition.

##### Biodiesel specification

Some physical and chemical properties of the produced biodiesel were determined under standard testing methods (ASTM). The methods followed for determining the specifications of the produced biodiesel were:

- ASTM D1298-12b^37^: determining the density at a reference temperature of 15 ºC.
- ASTM D240-17^38^: measuring the mass heat of combustion.
- ASTM D445-17a^39^: measuring the kinematic viscosity.
- ASTM D947-14e2^40^: determining the acid value.
- ASTM D93-16a^41^: measuring the flash point through Pensky-Martens closed cup tester.
- ASTM D7346-15^42^: measuring pour point.
- ASTM D2500-17^43^: measuring cloud point.
- Cetane number (CN):

In the present study, the CN was determined using Eq. (2)^44,45^:

2 $${\text{CN = }}\sum {{\text{ME}}_{{({\text{Wt}}\% )}} *{\text{ME}}_{{({\text{CN}})}} }$$

where CN: cetane number of the produced biodiesel; ME _(Wt%)_: weight percentage of each methyl ester; ME _(CN)_: cetane number of each methyl ester.

## Results and discussion

### Choice of the blend ratio of unrefined palm-WCO

The main factors considered for the blend fractions choice were the feedstock cost with its acid value, oxidative stability, cold flow properties, and the combustion quality of the produced biodiesel from it. If the biodiesel properties are poor, this may cause impairment to diesel engine performance and biodiesel quality^46,47^. The choice of the blend fractions was done by correlating the experimentally obtained results of cold flow properties of biodiesel produced from each blend prepared and the acid value of each blend. From an economic point of view as the most important parameter of the blend optimization, it was decided that the WCO should represent ≥ 50% of the blend to make the feedstock more cost-effective particularly for use on the industrial scale. Although the blends of ≥ 50% palm oil have lower acid content and higher oxidation stability, which are needed for good quality biodiesel, these blends were discarded. Hence the 50% palm:50% WCO and 40% palm:60% WCO blends were considered in terms of free fatty acid content of the blends before and after transesterification. Their acid values before transesterification were 7.8 and 8.4 mg KOH/g oil, respectively whereas after transesterification the values were 0.68 and 0.7 mg KOH/g FAME, respectively. Table 4 illustrates the cloud and pour points of the seven blends-based biodiesel. The biodiesel from blends ratio of (100:0, 80:20, and 60:40) palm oil to WCO wt%, showed relatively high PPs and CPs compared to the cold flow properties of the other blends. These high PPs and CPs are considered poor cold flow properties for biodiesel. The biodiesel can be used in areas of constant warm to hot climate throughout the year. Lastly, the (40% palm: 60% WCO) blend was selected because it has good cold flow properties, relatively low acid value, good oxidation stability as a result of the presence of a moderately high percentage of palm oil in the blend and above all of that, the blend is more cost-effective and serves more the concept of food security by reducing the palm oil as much as possible.

**Table 4:** Cloud and pour points of biodiesel produced through homogeneously catalyzed transesterification from unrefined palm-WCO blends at different ratios.

Palm oil : WCO, wt%	Cloud point, °C	Pour point, °C
100 : 0	17	12
80 : 20	13	8
60 : 40	10	4.5
50 : 50	6	2.5
40 : 60	2.5	1
20 : 80	1.5	0
0 : 100	- 2	- 3.5

### Regression models development

A quadratic model (Eq. 3) was selected due to its minimal difference between adjusted and predicted R^2^ values. P-values less than 0.05 indicated the model terms were statistically significant. The Lack of Fit P-value (0.72%) suggested a low probability that this result was due to noise, as shown in Table S-1. The response variable for the model was transesterification conversion, while independent variables included reaction time (1–6 h), methanol-to-oil molar ratio (12:1 to 18:1), catalyst loading (1–3 wt%), and temperature (45–65 °C), with experimental data detailed in Table S-2. The methodology for developing regression models and testing their significance followed that of Al-Sakkari et al.^23^. Table S-3 presents the coefficients of the dimensionless quadratic model (Eq. 3) and the significance tests for model terms. The model’s F-value of 9.40 indicates high significance, with a P-value of 0.01% suggesting a low chance of this result being due to noise. Significant terms include A, B, C, D, and B^2^, while the Lack of Fit F-value of 11.62 further implies that the model is robust, with only a 0.72% chance of noise causing such a high value.

3 $$y = a_{0} + \sum a_{i} z_{i} + \sum a_{ij} z_{i} z_{j} + \sum a_{ii} z_{i}^{2}$$

From Table S-3, the regression dimensionless equation of the quadratic model is presented by Eq. 4. To convert the dimensionless forms to the actual ones, relations between x_i_ and z_i_ used in Al‐Sakkari et al.^23^ were implemented.

4 $$y = 90.82 + 8.11 z_{1} + 10.22 z_{2} + 16.31 z_{3} + 5.61 z_{4} - 14.8 z_{2}^{2}$$

### Model validation and selection

**Fig. 2:**
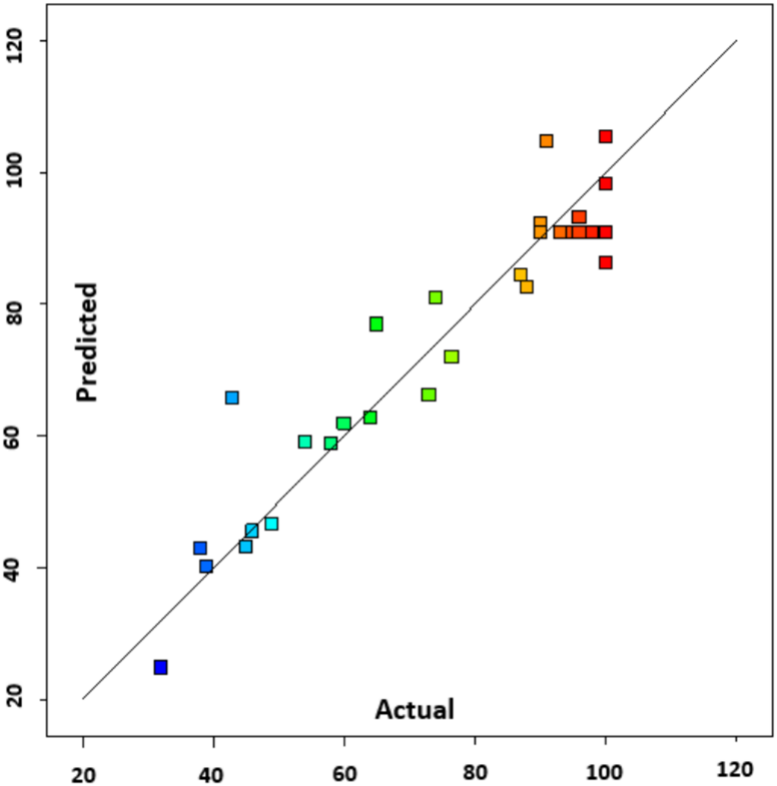
Predicted vs calculated conversion.

**Fig. 3:**
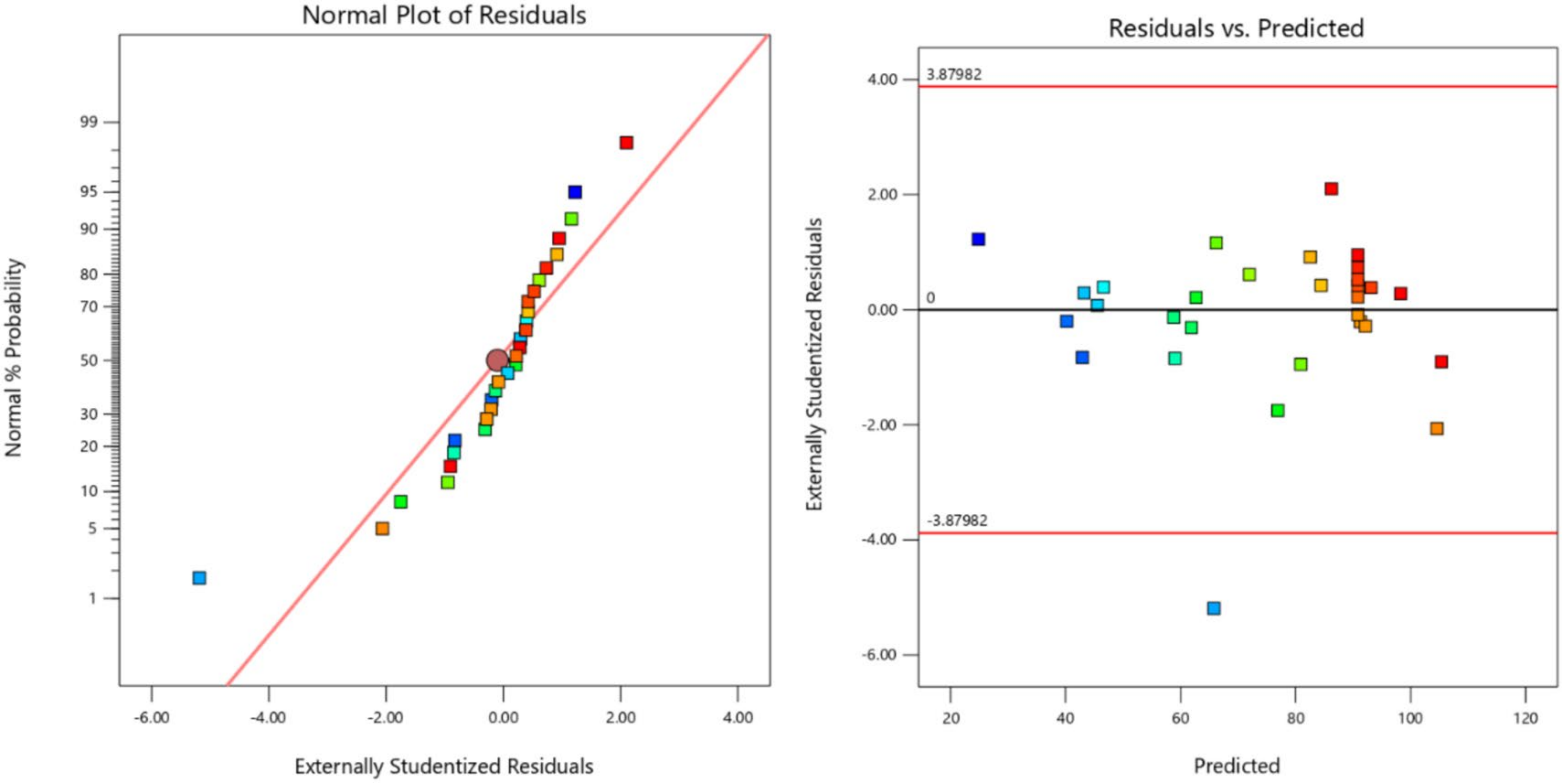
Residuals plots.

The determination coefficient (R^2^) was used as a tool for model validation as mentioned before in addition to the selection of the best model describing the relation between transesterification conversion and independent variables. To check the validity of the ANOVA test, the assumptions of the quadratic model must be normally distributed, the residuals must be randomly distributed, and the variance between the predicted and actual values must be minimal. Figure 2 presents the observed percentage conversion values versus the predicted ones using the quadratic model. Figure 3 shows the residuals plots, which indicates that the model is not biased due to the randomness of the data. Finally, coefficients of the uncoded quadratic model are tabulated in Table S-4.

### Effect of different conditions and their interactions on transesterification conversion

**Fig. 4:**
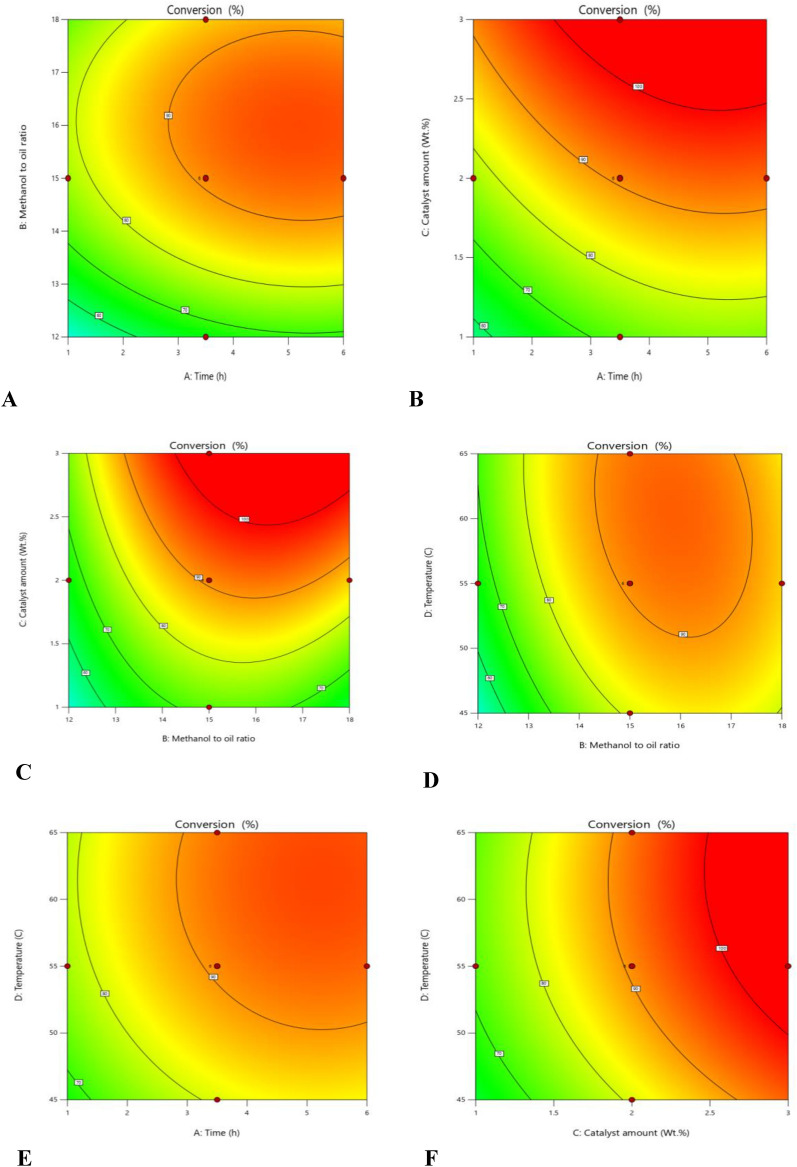
Contour plots of: (**A**) effect of molar ratio and time on conversion; (**B**) effect of catalyst loading and Time on conversion; (**C**) effect of catalyst loading and molar ratio on conversion; (**D**) effect of temperature and molar ratio on conversion; (**E**) effect of temperature and Time on conversion; (**F**) effect of Temperature and Catalyst loading on conversion.

Contour of percent conversion versus methanol to oil molar, catalyst loading, time, and temperature are shown in Fig. 4A–F. They show the effect of different conditions and their interactions on transesterification conversion. Generally, in the range of the present study, maximum values of conversion can be achieved at high values of molar ratio, time, catalyst loading, and temperature. As temperature increases, the reaction rate increases, and since transesterification is an endothermic reaction, the equilibrium conversion increases. In the case of catalyst loading, increasing the loading of the catalyst increases the available active sites, which results in increasing the percentage conversion of transesterification. The molar ratio has a positive effect on reaction conversion according to Le Chatelier’s principle, as the amount of reactants increases, the reaction proceeds in the forward direction. Time also has a positive effect on transesterification conversion and this is obviously observed from the quadratic models and the response surfaces in addition to the contour plots.

### 3D Surface response and conditions determination for near-complete transesterification conversion

**Fig. 5:**
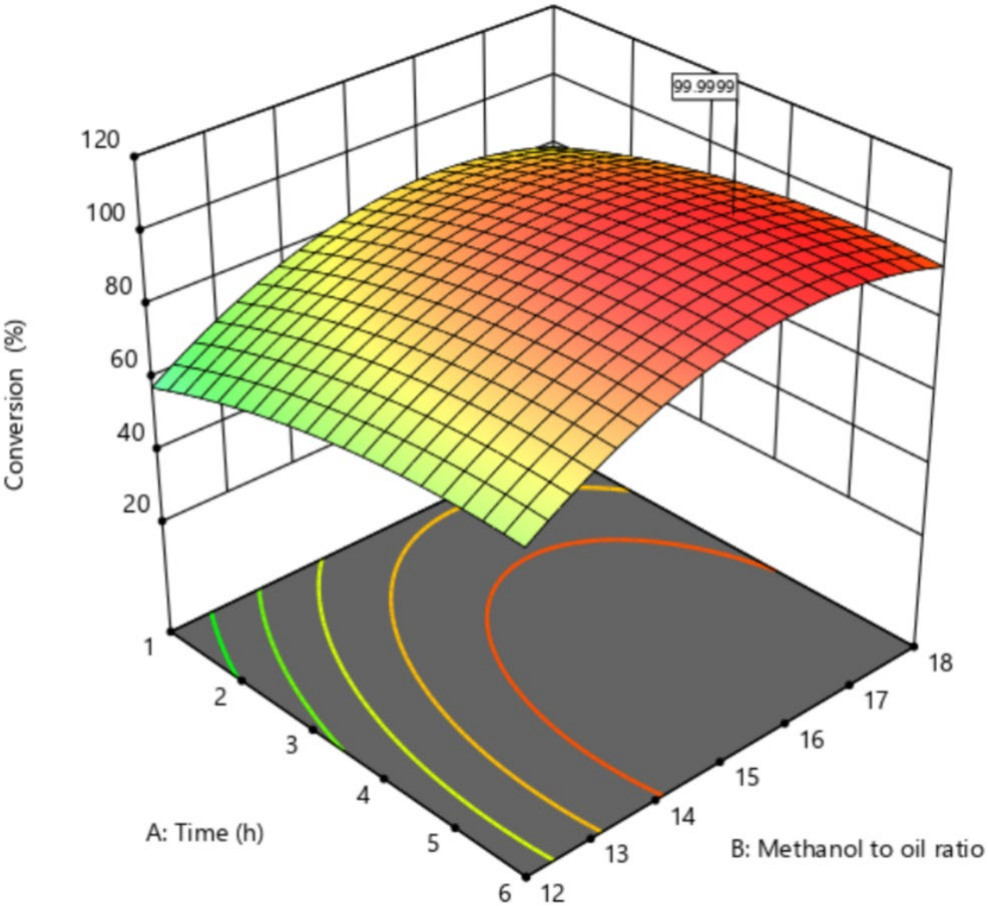
3D surface response of % conversion.

As previously mentioned, RSM was employed to identify the optimal conditions for the transesterification reaction and biodiesel production, aiming for near-complete conversion. By analyzing the response surfaces and contour plots, it was evident that multiple combinations of the selected independent variables could achieve this high conversion rate. A comprehensive list of these viable combinations is presented in Table S.5. Among them, the first solution, which deemed the most promising is illustrated through a 3D response surface in Fig. 5.

### Conditions determination for near complete conversion

As mentioned before, RSM was used to determine the conditions for transesterification reaction and biodiesel production for near complete conversion. Upon tracing the response surfaces and contour graphs, many different combinations of the selected independent variables can reach near complete conversion. Table S-5 shows a hundred points of these combinations. The main idea of different combinations is to give a variety of optimized set of parameters that give complete conversion of biodiesel that could work under different manufacturing or market challenges. For example, if there is a region that suffers from a high cost of energy, it is possible to get complete conversion using a temperature as low as 45.5 °C. If there is a high demand for biodiesel or a high labor cost, it is possible to get a complete conversion as fast as 1.72 h, …etc.

### Characterization of the produced biodiesel

Table 5 presents the results of characterization tests done to determine the characteristics of the produced biodiesel. The properties of the produced biodiesel meet the standard specifications of biodiesel. These good properties of biodiesel such as heat of combustion, cold flow properties, and viscosity lead to desirable diesel engine performance. The high flash point of biodiesel produced adds an important safety factor for using it. Also, the enhancement of cold flow properties achieved by mixing unrefined palm oil and WCO assures the possibility of using the produced biodiesel in cold weather (minimum 3 °C). Whereas the low acid content of the produced biodiesel can play an important role in avoiding corrosion of the internal parts of the engine. The acid value was about 0.7 mg KOH per gram of FAMEs produced.

**Table 5:** Characteristics of the produced biodiesel.

Property	Value	Standard
Viscosity 40 °C cSt	4.7	4.0–6.0
Density g/ml	0.86	0.86–0.9
Heat of combustion kJ/kg	38,140	35,000–42,000
Flash point °C	165	$\ge$ 130
Cloud point °C	3	− 3 to 12
Pour point °C	1	− 15 to 10
Acid value mg KOH/g FAMEs	0.7	≤ 0.8

Tables 6 and 7 summarize the GC analysis results of fatty acids composition of the two biodiesel samples produced through transesterification and the reaction conditions and considerations for producing these samples, respectively. From the analysis results, sample (1) has a higher saturated fatty acid content than that in sample (2) while it is lower in polyunsaturated fatty acid composition.

**Table 6:** Fatty acids composition of some produced biodiesel samples.

Fatty acids	Mass % of Sample (1)	Mass % of Sample (2)
Myristic acid C14	1.48	0.62
Palmitic acid C16	33.41	23.47
Stearic acid C18	2.30	3.62
Oleic acid C18:1	45.63	38.26
Linoleic A. C18:2	15.35	32.60
Linolenic A. C18:3	1.48	1.40
Saturated fatty A. (SFA)	37.19	27.71
Unsaturated F.A. (USFA)	62.46	72.26
Mono unsaturated F.A. (MUSFA)	45,63	38.26
Poly unsaturated F.A. (PUSFA)	16.83	34.00
Total F.A. (TFA)	99.65	99.97

**Table 7:** Transesterification considerations and conditions.

Catalyst	Hetero (CKD) 1	Hetero (CKD) 2
Catalyst loading	3%	2.7%
Methanol to feedstock oil molar ratio	15:1	17:1
Reaction time (h)	3.5	4.5
Reaction temperature (**°**C)	65	60
Conversion, molar basis	100%	100%

This indicates that sample (1) has higher oxidation stability than the other sample^46,48^.

Finally, the CN of the produced biodiesel was calculated using Eq. (2). The CN calculated for samples 1 and 2 were 61 and 57.5, respectively. This indicates that both biodiesel samples have good combustion quality and ignition properties that can reflect positively on diesel engine performance^49^. The higher cetane number of sample 1 may be a result of the higher content of saturated fatty acids such as palmitic acid^50^.

## Conclusions

The results of this study demonstrate the effectiveness of using waste CKD as a catalyst for the transesterification of mixed oil made from WCO and low-grade unrefined palm oil to produce biodiesel. The use of factorial design and response surface methodology allowed for the efficient

generation of data and the determination of the optimal conditions for CKD-catalyzed transesterification, considering factors such as the methanol to oil molar ratio, reaction time, catalyst loading, and reaction temperature. The developed quadratic model accurately predicted the range of experimental results. The resulting biodiesel, made from a blend of palm oil and WCO in a 2:3 ratio (40 wt% palm oil, 60 wt% WCO), showed good fuel properties and is suitable for various uses while contributing to the concepts of energy, food, and environmental securities. If calcinated probably, CKD demonstrated good catalytic performance and complete conversion at somehow reasonable reaction conditions, making it a potential candidate for commercial-scale biodiesel production due to its availability as a waste product in cement-producing countries and the possibility for reuse. Further research is recommended to improve efficiency and economic feasibility, such as studying reaction kinetics and substituting low- to moderate- grade palm oil with waste cooking palm oil. This could contribute to reducing the total production costs, while maintaining superior product characteristics.

## Supplementary Information

Supplementary Information.

## Data Availability

Enquiries about data availability should be directed to the corresponding author (Tamer S. Ahmed).
