# Supplementary material for: Optimized transesterification of unrefined palm and waste cooking oil blend to biodiesel using cement kiln dust catalyst
Source: Sci Rep. 2025 Jul 28;15:27397. doi: 10.1038/s41598-025-11752-x (PMC12304178; doi:10.1038/s41598-025-11752-x)
Supplement: Supplementary file 1 — Supplementary Information. [file 41598_2025_11752_MOESM1_ESM.docx]

Supporting Information

Cement Kiln Dust Catalyzed Transesterification of Unrefined Palm and Waste Cooking Oils Blend to Biodiesel with Optimized Parameters and Product Characterization

Mahmoud S. Hefney, Mai O. Abdelmigeed, Tamer S. Ahmed, Ibrahim M. Ismail

**Table S-1:** Summary of fit for various models.

| **Source** | **Sequential p-value** | **Lack of Fit p-value** | **Adjusted R²** | **Predicted R²** |  |
| --- | --- | --- | --- | --- | --- |
| Linear | 0.0003 | 0.0010 | 0.4908 | 0.3937 |  |
| 2FI | 0.9259 | 0.0005 | 0.3890 | -0.2587 |  |
| **Quadratic** | **0.0002** | **0.0072** | **0.8022** | **0.5707** | **Suggested** |
| Cubic | 0.0892 | 0.0134 | 0.9018 | -1.3345 | Aliased |

**Table S-2:** Matrix of independent variables and the response of different runs

| Type of point | x_1_ time h | x_2_ methanol to oil molar ratio | x_3_ Catalyst amount wt% | x_4_ Temperature C | Y Conversion% |
| --- | --- | --- | --- | --- | --- |
| Factorial | 6 | 18 | 3 | 65 | 100 |
| Factorial | 6 | 18 | 3 | 45 | 100 |
| Factorial | 6 | 18 | 1 | 65 | 60 |
| Factorial | 6 | 18 | 1 | 45 | 54 |
| Factorial | 6 | 12 | 3 | 65 | 88 |
| Factorial | 6 | 12 | 3 | 45 | 64 |
| Factorial | 6 | 12 | 1 | 65 | 58 |
| Factorial | 6 | 12 | 1 | 45 | 45 |
| Factorial | 1 | 18 | 3 | 65 | 90 |
| Factorial | 1 | 18 | 3 | 45 | 87 |
| Factorial | 1 | 18 | 1 | 65 | 46 |
| Factorial | 1 | 18 | 1 | 45 | 38 |
| Factorial | 1 | 12 | 3 | 65 | 73 |
| Factorial | 1 | 12 | 3 | 45 | 49 |
| Factorial | 1 | 12 | 1 | 65 | 39 |
| Factorial | 1 | 12 | 1 | 45 | 32 |
| Star | 6 | 15 | 2 | 55 | 96 |
| Star | 1 | 15 | 2 | 55 | 65 |
| Star | 3.5 | 15 | 3 | 55 | 91 |
| Star | 3.5 | 15 | 1 | 55 | 76.5 |
| Star | 3.5 | 18 | 2 | 55 | 100 |
| Star | 3.5 | 12 | 2 | 55 | 43 |
| Star | 3.5 | 15 | 2 | 65 | 90 |
| Star | 3.5 | 15 | 2 | 45 | 74 |
| Center | 3.5 | 15 | 2 | 55 | 100 |
| Center | 3.5 | 15 | 2 | 55 | 95 |
| Center | 3.5 | 15 | 2 | 55 | 96 |
| Center | 3.5 | 15 | 2 | 55 | 98 |
| Center | 3.5 | 15 | 2 | 55 | 93 |
| Center | 3.5 | 15 | 2 | 55 | 90 |

Table S-3: Coefficients of the dimensionless models and their significance tests ANOVA for quadratic model (S: Significant; I: Insignificant)

| **Source** | **Sum of Squares** | **df** | **Mean Square** | **F-value** | **p-value** |  | **Coeff.** | **Value** | **Res.** |
| --- | --- | --- | --- | --- | --- | --- | --- | --- | --- |
| **Model** | 13469.75 | 14 | 962.13 | 9.40 | < 0.0001 | S | a_0_ | 90.82 | S |
| A-Time | 1184.22 | 1 | 1184.22 | 11.57 | 0.0039 |  | a_1_ | 8.11 | S |
| B-Methanol to oil ratio | 1880.89 | 1 | 1880.89 | 18.38 | 0.0006 |  | a_2_ | 10.22 | S |
| C-Catalyst amount | 4785.68 | 1 | 4785.68 | 46.77 | < 0.0001 |  | a_3_ | 16.31 | S |
| D-Temperature | 566.72 | 1 | 566.72 | 5.54 | 0.0327 |  | a_4_ | 5.61 | S |
| AB | 5.06 | 1 | 5.06 | 0.0495 | 0.8270 |  | a_11_ | -5.80 | I |
| AC | 5.06 | 1 | 5.06 | 0.0495 | 0.8270 |  | a_22_ | -14.80 | S |
| AD | 0.0625 | 1 | 0.0625 | 0.0006 | 0.9806 |  | a_33_ | -2.55 | I |
| BC | 390.06 | 1 | 390.06 | 3.81 | 0.0698 |  | a_44_ | -4.30 | I |
| BD | 162.56 | 1 | 162.56 | 1.59 | 0.2268 |  | a_12_ | -0.5625 | I |
| CD | 18.06 | 1 | 18.06 | 0.1765 | 0.6803 |  | a_13_ | -0.5625 | I |
| A² | 87.11 | 1 | 87.11 | 0.8513 | 0.3708 |  | a_14_ | 0.0625 | I |
| B² | 567.38 | 1 | 567.38 | 5.55 | 0.0326 |  | a_23_ | 4.94 | I |
| C² | 16.82 | 1 | 16.82 | 0.1644 | 0.6908 |  | a_24_ | -3.19 | I |
| D² | 47.87 | 1 | 47.87 | 0.4678 | 0.5044 |  | a_34_ | 1.06 | I |
| **Residual** | 1534.82 | 15 | 102.32 |  |  |  |  |  |  |
| Lack of Fit | 1471.49 | 10 | 147.15 | 11.62 | 0.0072 | S |  |  |  |
| Pure Error | 63.33 | 5 | 12.67 |  |  |  |  |  |  |
| **Cor Total** | 15004.58 | 29 |  |  |  |  |  |  |  |

**Table S-4:** Coefficients of the uncoded quadratic model

| Term | Coefficient |
| --- | --- |
| Constant, a_0_ | -588.28371 |
| a_1_ | +11.17598 |
| a_2_ | +55.54948 |
| a_3_ | -3.24521 |
| a_4_ | +6.66168 |
| a_1_*a_1_ | -0.927719 |
| a_2_*a_2_ | -1.64425 |
| a_3_*a_3_ | -2.54825 |
| a_4_*a_4_ | -0.042982 |
| a_1_*a_2_ | -0.075000 |
| a_1_*a_3_ | -0.225000 |
| a_1_*a_4_ | +0.002500 |
| a_2_*a_3_ | +1.64583 |
| a_2_*a_4_ | -0.106250 |
| a_3_*a_4_ | +0.106250 |

**Table S-5:** Optimum conditions for different parameter combinations

| **Number** | **Time** | **Methanol to oil ratio** | **Catalyst amount** | **Temperature** | **Conversion** |
| --- | --- | --- | --- | --- | --- |
| 1 | 3.11 | 17.22 | 2.59 | 63.54 | 100 |
| 2 | 4.05 | 16.39 | 2.33 | 56.16 | 100 |
| 3 | 5.68 | 17.71 | 2.97 | 45.50 | 100 |
| 4 | 4.59 | 16.72 | 2.45 | 51.33 | 100 |
| 5 | 3.96 | 16.61 | 2.63 | 49.10 | 100 |
| 6 | 5.20 | 15.97 | 2.46 | 50.83 | 100 |
| 7 | 3.23 | 17.00 | 2.63 | 52.30 | 100 |
| 8 | 5.53 | 16.68 | 2.26 | 61.10 | 100 |
| 9 | 1.89 | 16.02 | 2.84 | 58.62 | 100 |
| 10 | 3.05 | 15.61 | 2.77 | 51.46 | 100 |
| 11 | 2.22 | 15.97 | 2.80 | 55.14 | 100 |
| 12 | 4.97 | 14.81 | 2.35 | 64.54 | 100 |
| 13 | 2.17 | 17.81 | 2.98 | 63.57 | 100 |
| 14 | 3.14 | 15.65 | 2.59 | 54.27 | 100 |
| 15 | 5.96 | 13.63 | 2.95 | 64.28 | 100 |
| 16 | 2.67 | 16.19 | 2.55 | 62.04 | 100 |
| 17 | 2.97 | 15.09 | 2.58 | 62.71 | 100 |
| 18 | 3.47 | 15.87 | 2.94 | 47.33 | 100 |
| 19 | 2.79 | 16.22 | 2.59 | 55.47 | 100 |
| 20 | 3.67 | 14.49 | 2.69 | 57.43 | 100 |
| 21 | 4.72 | 14.74 | 2.47 | 56.46 | 100 |
| 22 | 2.99 | 16.77 | 2.81 | 49.46 | 100 |
| 23 | 3.28 | 16.67 | 2.44 | 58.06 | 100 |
| 24 | 2.50 | 16.27 | 2.79 | 52.65 | 100 |
| 25 | 5.69 | 15.97 | 2.23 | 63.71 | 100 |
| 26 | 3.24 | 17.04 | 2.50 | 57.06 | 100 |
| 27 | 2.87 | 16.98 | 2.56 | 59.49 | 100 |
| 28 | 3.95 | 14.25 | 2.81 | 56.36 | 100 |
| 29 | 1.72 | 16.77 | 3.00 | 54.59 | 100 |
| 30 | 5.31 | 16.51 | 2.24 | 62.44 | 100 |
| 31 | 1.72 | 16.70 | 2.96 | 55.72 | 100 |
| 32 | 5.62 | 15.83 | 2.23 | 63.64 | 100 |
| 33 | 5.50 | 15.29 | 2.25 | 63.73 | 100 |
| 34 | 2.36 | 16.28 | 2.66 | 63.63 | 100 |
| 35 | 5.66 | 16.44 | 2.27 | 63.79 | 100 |
| 36 | 4.38 | 15.69 | 2.35 | 54.52 | 100 |
| 37 | 3.30 | 14.12 | 2.86 | 63.17 | 100 |
| 38 | 1.63 | 15.83 | 2.95 | 60.52 | 100 |
| 39 | 4.72 | 14.88 | 2.42 | 56.59 | 100 |
| 40 | 5.28 | 16.92 | 2.29 | 60.99 | 100 |
| 41 | 4.60 | 14.80 | 2.92 | 49.75 | 100 |
| 42 | 2.92 | 16.96 | 2.55 | 60.26 | 100 |
| 43 | 3.47 | 15.20 | 2.43 | 62.81 | 100 |
| 44 | 5.14 | 16.79 | 2.52 | 49.90 | 100 |
| 45 | 3.58 | 16.32 | 2.58 | 51.09 | 100 |
| 46 | 5.02 | 13.78 | 2.78 | 64.73 | 100 |
| 47 | 5.78 | 15.31 | 2.27 | 64.06 | 100 |
| 48 | 2.62 | 15.91 | 2.88 | 51.07 | 100 |
| 49 | 2.76 | 15.57 | 2.97 | 50.23 | 100 |
| 50 | 3.17 | 16.07 | 2.86 | 48.68 | 100 |
| 51 | 5.36 | 15.18 | 2.26 | 63.65 | 100 |
| 52 | 3.14 | 16.89 | 2.72 | 50.37 | 100 |
| 53 | 5.36 | 14.44 | 2.52 | 57.85 | 100 |
| 54 | 4.61 | 17.77 | 2.53 | 62.79 | 100 |
| 55 | 4.12 | 16.04 | 2.26 | 60.62 | 100 |
| 56 | 5.69 | 14.00 | 2.74 | 59.10 | 100 |
| 57 | 3.03 | 16.74 | 2.49 | 59.25 | 100 |
| 58 | 5.33 | 15.08 | 2.27 | 62.86 | 100 |
| 59 | 5.43 | 16.76 | 2.30 | 55.89 | 100 |
| 60 | 4.30 | 13.97 | 2.87 | 57.67 | 100 |
| 61 | 5.45 | 16.53 | 2.27 | 64.01 | 100 |
| 62 | 3.19 | 15.20 | 2.64 | 55.09 | 100 |
| 63 | 2.83 | 16.25 | 2.62 | 54.04 | 100 |
| 64 | 3.21 | 17.89 | 2.70 | 57.37 | 100 |
| 65 | 3.69 | 15.65 | 2.71 | 50.00 | 100 |
| 66 | 3.64 | 17.16 | 2.52 | 53.92 | 100 |
| 67 | 4.93 | 15.43 | 2.40 | 53.52 | 100 |
| 68 | 5.75 | 14.46 | 2.45 | 61.89 | 100 |
| 69 | 2.01 | 15.93 | 2.84 | 56.74 | 100 |
| 70 | 4.71 | 15.39 | 2.64 | 49.92 | 100 |
| 71 | 2.37 | 17.47 | 2.99 | 50.80 | 100 |
| 72 | 2.03 | 17.35 | 2.91 | 55.71 | 100 |
| 73 | 4.63 | 17.39 | 2.47 | 53.57 | 100 |
| 74 | 5.86 | 16.76 | 2.32 | 63.78 | 100 |
| 75 | 4.70 | 15.62 | 2.23 | 58.68 | 100 |
| 76 | 2.50 | 15.88 | 2.78 | 53.59 | 100 |
| 77 | 3.00 | 15.89 | 2.57 | 54.82 | 100 |
| 78 | 2.20 | 14.68 | 2.99 | 62.82 | 100 |
| 79 | 5.09 | 17.08 | 2.60 | 48.86 | 100 |
| 80 | 3.99 | 15.44 | 2.33 | 64.39 | 100 |
| 81 | 3.91 | 15.52 | 2.48 | 53.63 | 100 |
| 82 | 4.52 | 16.03 | 2.70 | 47.77 | 100 |
| 83 | 2.87 | 16.31 | 2.81 | 50.16 | 100 |
| 84 | 3.13 | 14.56 | 2.69 | 62.44 | 100 |
| 85 | 3.44 | 17.08 | 2.68 | 50.28 | 100 |
| 86 | 5.34 | 15.15 | 2.52 | 52.51 | 100 |
| 87 | 5.55 | 15.21 | 2.43 | 53.90 | 100 |
| 88 | 5.88 | 15.98 | 2.24 | 63.50 | 100 |
| 89 | 5.73 | 14.27 | 2.53 | 61.62 | 100 |
| 90 | 4.38 | 16.63 | 2.75 | 46.82 | 100 |
| 91 | 5.37 | 15.46 | 2.26 | 57.44 | 100 |
| 92 | 5.36 | 14.95 | 2.51 | 53.71 | 100 |
| 93 | 5.89 | 14.27 | 2.54 | 64.08 | 100 |
| 94 | 3.00 | 14.62 | 2.75 | 59.37 | 100 |
| 95 | 5.29 | 16.82 | 2.27 | 59.61 | 100 |
| 96 | 4.37 | 17.70 | 2.92 | 46.05 | 100 |
| 97 | 5.76 | 16.06 | 2.22 | 59.46 | 100 |
| 98 | 3.51 | 17.36 | 2.67 | 51.10 | 100 |
| 99 | 3.21 | 16.93 | 2.76 | 49.50 | 100 |
| 100 | 2.41 | 14.96 | 2.80 | 62.28 | 100 |


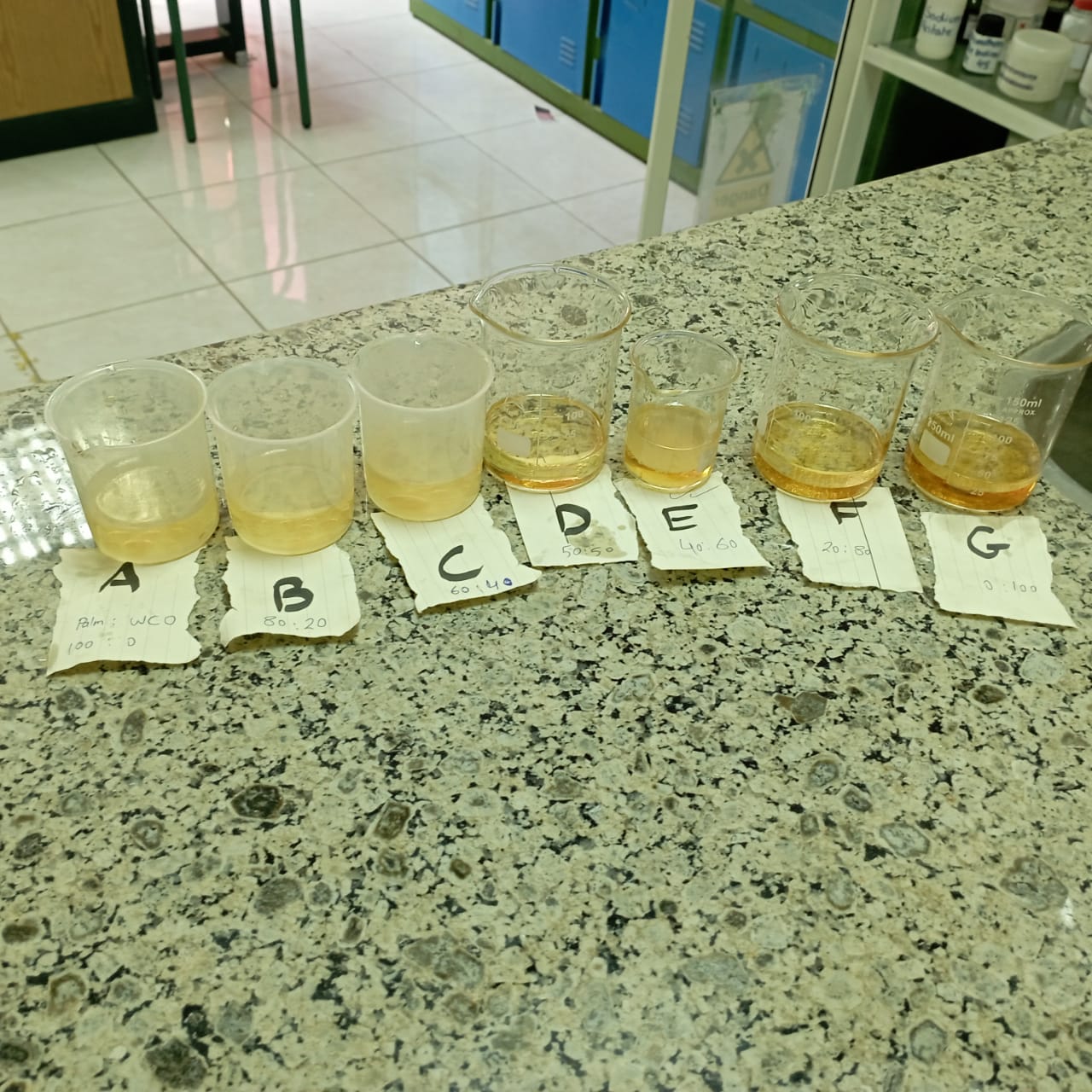


Figure (S-1): Seven samples of different palm oil to WCO weight percentage ratios: A) 100:0, B) 80:20, C) 60:40, D) 50:50, E) 40:60, F) 20:80, and G) 0:100.
(Sample E was chosen to be the used blend)
